# Unlocking the Diagnostic Potential: A Systematic Review of Biomarkers in Spinal Tuberculosis

**DOI:** 10.3390/jcm13175028

**Published:** 2024-08-25

**Authors:** Andre Marolop Pangihutan Siahaan, Alvin Ivander, Steven Tandean, Rr. Suzy Indharty, Eric Teo Fernando, Stefanus Adi Nugroho, Viria Milenia, Dhea Olivia Az Zahra

**Affiliations:** 1 Department of Neurosurgery, Faculty of Medicine, Universitas Sumatera Utara, Medan 20155, Indonesia; steven.tandean@usu.ac.id (S.T.); rr.suzyi@usu.ac.id (R.S.I.); 2 Center of Evidence Based Medicine, Faculty of Medicine, Universitas Sumatera Utara, Medan 20155, Indonesia; alvinivander@petalmail.com (A.I.); eric.teo1234@gmail.com (E.T.F.); nstefanusadi@gmail.com (S.A.N.); milenconan00@gmail.com (V.M.); dhea.olive@gmail.com (D.O.A.Z.)

**Keywords:** spinal tuberculosis, biomarker, diagnostic, clinical

## Abstract

**Background/Objectives**: Spinal tuberculosis (STB) is frequently misdiagnosed due to the multitude of symptoms it presents with. This review aimed to investigate the biomarkers that have the potential to accurately diagnose spinal TB in its early stages. **Methods**: A systematic search was conducted across multiple databases, yielding a diverse range of biomarkers categorized into complete blood count parameters, host inflammatory responses, bacterial antigens, and RNA-based markers. This review included studies on spinal tuberculosis patients, including blood serum biomarkers, while exclusion criteria included pediatric cases, cerebrospinal fluid or imaging biomarkers, co-infection with other bacteria, viruses, comorbidities, tumors, immune diseases, HIV infection, metabolic disorders, animal studies, opinion papers, and biomarkers relevant to health problems outside the disease. QUADAS-2 was used as a quality assessment tool for this review. This review identifies several promising biomarkers with significant diagnostic potential. **Results**: The neutrophil-to-lymphocyte ratio (NLR), monocyte-to-lymphocyte ratio (MLR), IFN-γ, CXCR3, CXCL9, CXCL10, PSMB9, STAT1, TAP1, and specific miRNA combinations demonstrated noteworthy diagnostic accuracy in distinguishing STB from other spinal pathologies. Additionally, these biomarkers offer insights into disease severity and progression. The review also highlighted the importance of combining multiple biomarkers to enhance diagnostic precision. This comprehensive systematic review underscores the potential of biomarkers to revolutionize the diagnosis of spinal tuberculosis. By integrating these markers into clinical practice, healthcare providers can achieve earlier and more accurate diagnosis, leading to improved patient care and outcomes. **Conclusions**: The combination of multiple biomarkers, including NLR, PSMB9, STAT1, and specific miRNAs, demonstrates promising diagnostic accuracy.

## 1. Introduction

Spinal tuberculosis (STB) cases are frequently missed and misdiagnosed due to the wide range of symptoms that mimic other spinal diseases [1]. Untreated cases can cause complications, such as permanent neurological deficits and spinal deformities [2,3]. Though STB accounts for only 2% of all TB cases, the increasing trend in overall TB incidence is expected to increase the number of STB cases. Therefore, early diagnosis and prompt treatment are needed to prevent permanent disability, minimize spinal deformities, and reduce economic burden [2,4,5].

To date, various diagnostic modalities have been employed for STB, including imaging techniques, laboratory investigations, and tissue diagnosis methods such as staining, culture, molecular diagnostics, PCR, and conventional histopathology [3]. Every tool has its own advantages and limitations. The definitive diagnostic techniques for STB are invasive. However, these tests yielded low positive results. Acid-fast bacilli staining (AFBS) produced positive results in only 38% of patients. In contrast, culture identified 50% of cases, and histopathology identified 60% of cases. It has been observed that imaging modalities and laboratory examinations do not possess the ability to distinguish among STB, pyogenic spinal infection, and spinal neoplasm [3,6]. Given the limitations of the existing tools, more precise and effective diagnostic tools are required.

In recent years, there has been a global push for research on biomarkers, resulting in notable advancements [7,8]. Biomarkers (biological markers) are characteristics that are measured as indicators of normal biological processes, pathogenic processes, or biological responses to an exposure or intervention, including therapeutic interventions [9]. Extensive research has been conducted on biomarkers for pulmonary tuberculosis (TB), revealing studies that have demonstrated significant diagnostic potential. There is little knowledge on biomarkers for the diagnosis of STB, but this may show potential [1]. Hence, the primary objective of this systematic study was to investigate the biomarkers that have the potential to accurately diagnose spinal TB in its early stages.

## 2. Materials and Methods

### 2.1. Study Design

This study is a systematic review that was designed using the Preferred Reporting Items for Systematic Reviews and Meta-Analyses (PRISMA) [10]. The objective of this study was to review the biomarkers that have been investigated for diagnostic purposes, their accuracy among patients with suspected or confirmed spinal tuberculosis, and how they compare to established diagnostic methods. The key elements of the research question were as follows: suspected or confirmed spinal tuberculosis (patient), biomarkers to diagnose spinal tuberculosis (index test), and the use of histopathology and culture (reference standard). Our study was registered in PROSPERO (National Institute for Health and Care Research, York, UK) under ID CRD42023472075. 

### 2.2. Data Collection

Search strategies were designed in collaboration with a librarian based on keywords related to “spinal tuberculosis”, “biomarkers”, and “diagnostics”. Based on the selected keywords, a literature search was conducted using the electronic databases Web of Science, PubMed, and SCOPUS. The search was limited to articles published between 2013 and 2023, encompassing a period of ten years, to locate articles that were pertinent to the topic.

Keywords may be adjusted to align with the regulations of each database, thereby enhancing the literature search process. The criteria for inclusion of studies in this review were diagnostic studies with suspected or confirmed spinal tuberculosis patients, with or without tuberculosis in other organs, and studies examining biomarkers using blood serum as the source of detection. The exclusion criteria were as follows: diagnostic studies with cases of suspected or confirmed spinal tuberculosis in pediatric patients; studies examining biomarkers using cerebrospinal fluid (CSF) or imaging as the source of detection; case studies with co-infection with other bacteria, viruses, and comorbidities; co-occurrence of other tumors, immune diseases, neoplastic diseases, HIV infection, or metabolic disorders; animal studies; opinion papers; and studies of biomarkers relevant to health problems outside the spinal tuberculosis disease.

Three independent researchers used the eligibility criteria described and screened the titles and abstracts of the studies gathered from the search. The full text of the selected studies was reviewed to confirm their eligibility.

### 2.3. Quality Assessment

In line with the study objectives, we employed the QUADAS-2 (Quality Assessment of Diagnostic Accuracy Studies) (Cochrane, London, UK) tool to systematically assess the risk of bias and evaluate concerns related to the applicability of the selected literature in our research. This tool provides a systematic framework for assessing four key domains: patient selection, index tests, reference standards, and flow and timing [11]. QUADAS-2 is one of the most well-known tool for quality assessment in systematic review. While QUADAS-2 is not overly restrictive as to exclude studies with acceptable conclusion, it is rigorous enough to reveal low-quality study. Most studies regarding biomarkers in STB were plagued by problem such as the lack of an index-test and occasional difficulties in providing representative groups of patient. As such, QUADAS-2 was chosen by authors. Disagreements were resolved by discussion, and if still unresolved, the most senior reviewers adjudicated by considering all authors, reviewers, and the manuscript itself.

## 3. Results

### 3.1. Search Results

**Figure 1:**
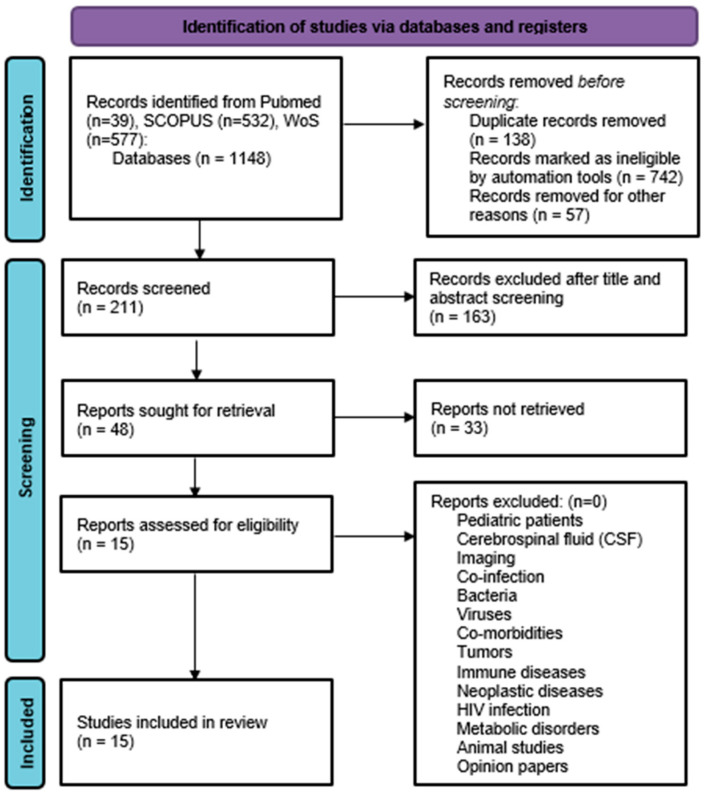
PRISMA flow chart.

An initial online search yielded a total of 1148 articles (39 articles in PubMed, 532 articles in SCOPUS, and 577 articles in WoS) that could potentially be relevant. After eliminating duplicate records and excluding records after screening, 48 articles were selected for the full-text examination. Upon conducting a thorough assessment of the eligibility of the full texts, 15 papers fulfilled the inclusion criteria and were incorporated into the review, as shown in Figure 1. 

### 3.2. Study Characteristic and Demographics

A majority of the studies were conducted in China (12/15; 80%), followed by South Africa (2/15; 13.3%), and Indonesia (1/15; 6.7%). The studies were published mainly between 2022 and 2023 (9/15; 60%), followed by 2020 (3/15; 20%), 2021 (2/15; 13.3%), and 2013 (1/15; 6.7%), indicating recent developments in this topic. Blood samples were mostly used in the studies (10/15; 66.7%), followed by tissue biopsy (5/15; 33.3%). Seven studies analyzed the host inflammatory response, three IHC for miRNA, three routine blood counts, and two bacterial antigens. The study characteristics and patient demographics are presented in Table 1.

### 3.3. Assessment of Risk of Bias and Applicability

**Figure 2:**
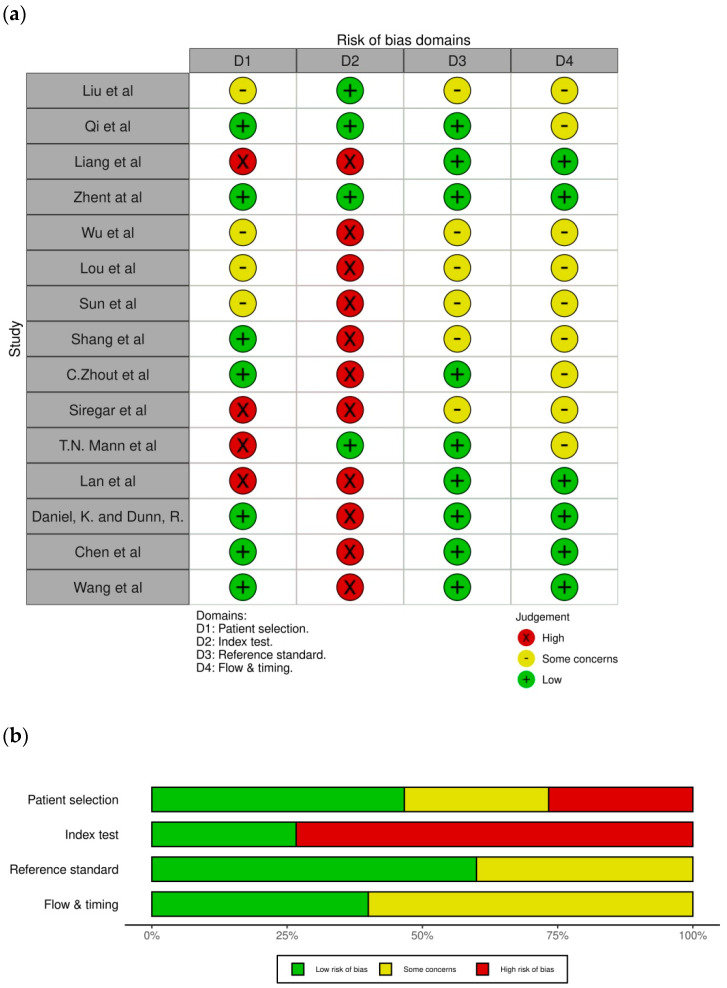
Risk of bias assessment using QUADAS-2. (**a**) Risk of bias assessment in respective studies; (**b**) Summary of assessment [1,5,6,7,8,12,13,14,15,16,17,18,19,20,21].

The results of the risk assessment of bias and applicability using QUADAS-2 showed that most studies had a lower risk of bias and were less concerned about the credibility of their findings. Only one study matched all the requirements in all domains [7]. The results of the risk of bias for all the studies in each domain are shown in Figure 2. The total risk of bias for each requirement was analyzed in all domains for the 15 selected studies, as shown in Figure 2. Approximately 27% of the studies in domain 1 had a high risk of bias in patient selection because of the lack of randomization in recruiting participants and the use of case–control design. In total, 27% of the studies in domain 2 had a low risk of bias in the index test, 60% of the studies in domain 3 had a low risk of bias in the reference test, and 40% of the studies in domain 4 had a low risk of bias in the flow and timing.

**Figure 3:**
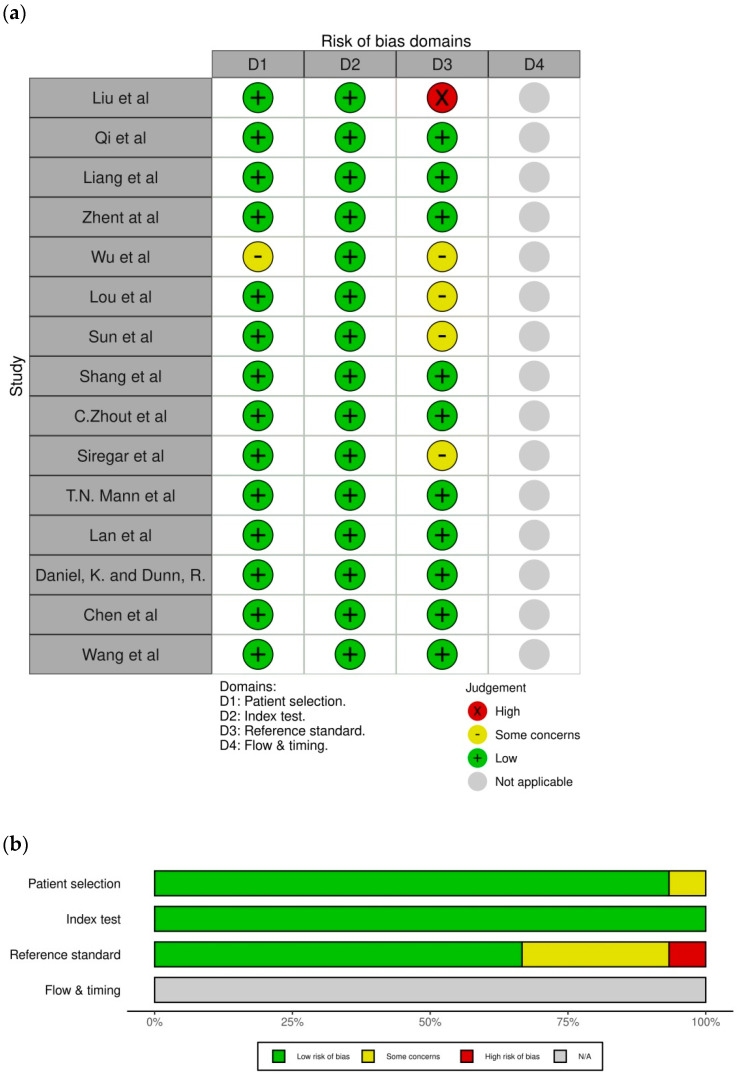
Applicability assessment using QUADAS-2. (**a**) Applicability assessment in respective studies; (**b**) Summary of assessment [1,5,6,7,8,12,13,14,15,16,17,18,19,20,21].

Most studies match all the requirements in all domains to assess the applicability of the studies. The applicability results of the studies in each domain are shown in Figure 3. The total risk of bias in the applicability of the studies for each requirement was analyzed in all domains for the 15 selected studies, as shown in Figure 3. Most studies have a low risk of bias in their applicability. Approximately 93% of the studies in domain 1 had a low risk of bias in patient selection, all of the studies in domain 2 had a low risk of bias in the index test, and 66% of the studies in domain 3 had a low risk of bias in the reference standard. The reference standard findings for all studies were evaluated without considering the index test results. The proper reference standard was used because all patients were included in the analysis

### 3.4. Biomarker Categorization

#### 3.4.1. Blood Cell Ratio and Complete Blood Count Parameters

Neutrophils possess the ability to impede the functioning of the immune system by suppressing lymphocyte activity and T-cell responses. A higher NLR can indicate increased inflammation or stress in the body. This ratio can be elevated in various conditions including infections, inflammation, autoimmune diseases, and certain types of cancer [22]. The NLR (AUC 0.87; 95% CI 0.75–0.87) exhibited good diagnostic efficacy in differentiating between STB and PSI with 78.33% sensitivity and 83.56% specificity. NLR could indeed serve as a useful marker for distinguishing individuals with STB from patients with PSI, as concluded from this study [5]. However, more studies on NLR are necessary to create a proper aggregation of biomarkers ability.

Tuberculosis infection has been observed to induce hematological alterations, including changes in monocyte and lymphocyte counts. Monocytes are proficient phagocytes that possess advanced capabilities in defense against various infections such as tuberculosis. Monocytes play a crucial role in the innate immune response and act as a link to the adaptive immune system through antigen presentation to lymphocytes [23]. MLR was used as a predictive tool for the diagnosis of STB and disease severity. The study found a significant positive correlation between MLR and the severity of STB, indicating that patients with higher MLR values tended to have more severe forms of the disease [20].

Thrombocytosis can be caused by various conditions, but systemic inflammation in response to infectious and inflammatory conditions is the most common. STB is associated with a higher platelet count than other spinal pathologies (*p* < 0.001). Thrombocytosis in STB is typically a reactive response to infection rather than a direct consequence of the tuberculosis itself. Unfortunately, elevated platelets had a sensitivity and specificity of 52.5% and 86.2%, respectively, for diagnosing STB [19].

#### 3.4.2. Immunoproteasome

PSMB9 is an immunoproteasome subunit, and its expression is upregulated in response to inflammatory stimuli. STAT1 is a signal transducer and activator that may be triggered by ligands including interferon-alpha and interferon-gamma. It is known to play a crucial role in the immune response against viral, fungal, and mycobacterial infections. TAP1 and PSMB9 play crucial roles in the assembly of heterodimer transporters and immune proteasomes. When TAP does not function properly, it can evade immune surveillance by pathogenic microorganisms. PSMB9, STAT1, and TAP1 levels were significantly correlated with activated dendritic cells, gamma delta T cells, immature B cells, and neutrophils. PSMB9, STAT1, and TAP1 may play key roles in the pathogenesis of TB, including spinal TB, and the protein products of these genes can serve as diagnostic markers and potential therapeutic targets for TB. The expression of these genes was found to be particularly high in patients with spinal TB and other forms of extrapulmonary TB (*p* < 0.05) [12].

The proteins MMP-9 (matrix metallopeptidase-9) and STAT1 (signal transducer and activator of transcription 1) have been associated with the degradation of discs in STB. MMP-9 is classified as a zinc-dependent endopeptidase, playing a crucial role in many tissue remodeling processes, both in physiological and pathological contexts [24]. On the other hand, STAT1 is a transcription factor expressed by the STAT1 gene in humans. According to a study conducted by Zhou et al., MMP-9 and STAT1 were associated with monocytes, neutrophils, and lymphocytes in investigations pertaining to immune cell infiltration. Determination of sexually transmitted bacterial infections (STBs) can be facilitated by assessing the immunological status of individuals. Immunohistochemistry findings indicated that there was a statistically significant increase in MMP-9 and STAT1 positivity among patients with STB (*p* < 0.01). Consistent with the findings of Zhou et al., a study conducted by Siregar et al. demonstrated the importance of utilizing serum MMP-9 in the context of STB.

#### 3.4.3. IFN-y, CXCR3, and Its Ligands (CXCL9 and CXCL10)

Interferon-gamma (IFN-gamma) is a glycoprotein with a molecular weight ranging from 20 to 25 kDa that is predominantly secreted by T-cells and natural killer (NK) cells in response to a diverse array of stimuli. IFN-γ exerts multifaceted effects on central nervous system (CNS) cells. CXCR3 is a G protein-coupled transmembrane receptor. It selectively binds to CXCL9, CXCL10, and CXCL11. CXCR3 is predominantly expressed in activated T lymphocytes and natural killer cells. The expression of IFN-γ, CXCR3, and its ligand was significantly upregulated in lesion tissue. Furthermore, with the use of ELISA, elevated levels of these molecules were detected in the peripheral blood of patients with ST in comparison to healthy controls [15].

#### 3.4.4. Fibrinogen, CRP, IFN-Gamma, NCAM, Ferritin, CXCL8, and GDF-15

Fibrinogen, CRP, IFN-gamma, NCAM, ferritin, CXCL8, and GDF-15 are well-recognized as significant biomarkers employed in both clinical and scientific contexts for the purpose of monitoring many facets of health, including inflammation, immunological response, and the evolution of diseases. Mann et al. [1], conducted a study examining the potential diagnostic use of fibrinogen, CRP, IFN-γ, NCAM, ferritin, CXCL8, and GDF-15 as host serum biomarkers for the diagnosis of STB. Fibrinogen, C-reactive protein (CRP), interferon-gamma (IFN-g), and neural cell adhesion molecule (NCAM) demonstrated the most significant discriminatory capacity as individual markers, as evidenced by their high area under the curve (AUC) values ranging from 0.88 to 0.99 on the receiver operating characteristic (ROC) plot. The study additionally showed the potential of CRP, NCAM, ferritin, CXCL8, and GDF-15 as biosignatures for STB, which could enhance diagnostic precision by utilizing a multi-biomarker approach. The sensitivity of the five-biomarker signature was 100%, with a 95% confidence interval (CI) ranging from 89% to 100%. Similarly, the specificity was 100%, with a 95% CI ranging from 84% to 100%. The receiver operating characteristic (ROC) plot area under the curve (AUC) was calculated to be 1.00, indicating perfect discrimination, with a 95% CI ranging from 1.00 to 1.00 [1].

#### 3.4.5. ANGPTL-4 (Angiopoietin-like Protein 4)

ANGPTL-4 (angiopoietin-like protein 4) is a special type of secreted protein involved in many biological processes, such as chronic inflammation, angiogenesis, and adjustment of vascular permeability. Higher expression of ANGPTL-4 is in line with increased formation of new capillaries, which can be found in STB patient compared to brucellosis infection [18]. This is associated with the major pathological changes in TB, namely, caseous necrosis and granulomas [25].

#### 3.4.6. Classically Activated Macrophages (M1) and Alternatively Activated (M2)

Classically activated macrophages (M1) and alternatively activated macrophages (M2) are macrophage polarizations that play different roles in the immune process. While M1 has a role in proinflammatory, M2 has an anti-inflammatory effect [26]. Wang et al. reported that both types of macrophages were present in tuberculous granulomas of STB patients, causing bone destruction [21].

#### 3.4.7. Lipopolysaccharide-Binding Protein (LBP)

Lipopolysaccharide-binding protein (LBP) is a 60 k Da molecular protein that belongs to type I reactive proteins and is produced during the acute phase of gram-negative bacterial infections in the peripheral blood [27]. The study conducted by Lou et al. revealed a statistically significant increase in LBP among patients with STB compared to individuals with no such condition (*p* < 0.001). The researcher also observed a statistically significant correlation between LBP protein expression and positive pathology, bacteriological findings, T-SPOT results, imaging diagnostic, and acid-resistance staining (*p* < 0.01) [13].

#### 3.4.8. Bacterial Antigen: Mycobacterium Tuberculosis-Specific Antigen/Phytohemagglutinin (TBAg/PHA) Ratio

Numerous researchers have asserted that the utilization of TBAg/PHA as a biomarker exhibits enhanced efficacy in discerning tuberculosis (TB) cases from individuals without the disease, as well as in distinguishing between current TB and latent TB infections [28,29,30]. A study conducted on STB patients showed consistent results. The diagnostic model developed by Qi et al. demonstrated improved predictive performance when using TBAg/PHA alone [6]. The model achieved area under the curve (AUC) values of 0.826 and 0.865 in the training and validation cohorts, respectively. According to the findings of Qi et al., the combination of the TBAg/PHA ratio with GeneXpert MTB/RIF demonstrated enhanced diagnostic efficacy for STB, as indicated by the respective area under the curve (AUC) values of 0.871 and 0.912 [6].

#### 3.4.9. RNA

For the combination of hsa-miR-506-3p, hsamiR-543, and hsa-miR-195-5p, the highest NPV was 78.38%. These tools also had the highest PPV values at 86.36%. In summary, the study concluded that the most powerful cutoff value to discriminate the two groups was 0.6156. The samples were divided into high- and low-risk categories using a cutoff criterion. The receiver operating characteristic (ROC) curve showed that the STB diagnostic model, which uses three miRNA biosignatures, is accurate. The AUC was 0.9020, demonstrating strong discrimination. Additionally, the model has a sensitivity of 87.9% and a specificity of 76.0%, indicating its ability to distinguish positive and negative situations [8].

The miRNA gene MIR125B1 is an RNA gene [31]. The area under the ROC curve (AUC) was 0.881, with a 95% confidence interval of 0.729–1.000. Sun et al. showed that miR-125b-5p in macrophage-derived exosomes from the tuberculosis-infected bone microenvironment may be a useful spinal tuberculosis diagnostic biomarker [14].

Nuclear-enriched abundant transcript 1 (NEAT1) is a long non-coding RNA (lncRNA) present at high levels in the nucleus of mammalian cells. Two distinct subtypes of long non-coding RNA NEAT1 exist that are specifically referred to as NEAT1_1 with a length of 3.7 kilobases and NEAT1_2 with a length of 23 kilobase [32]. Zheng et al. reported that NEAT1 lncRNA expression was dramatically increased in the peripheral blood and tissues of Mtb-infected STB and THP-1 cells. Paraspinal abscesses, >3 segments of the lesions, and 2 weeks of anti-TB medication were strongly associated with increased NEAT1 lncRNA expression in the peripheral blood of patients with STB. Additionally, STB patients peripheral blood serum lncRNA NEAT1 expression was strongly linked with IL-6, CRP, and ESR [7].

## 4. Discussion

This systematic study aimed to investigate a comprehensive array of biomarkers associated with the diagnosis of spinal tuberculosis (STB). These biomarkers are classified into many categories, including complete blood count measures, host inflammatory responses, bacterial antigens, and RNA-based markers. Significant among these discoveries was the diagnostic capabilities of the neutrophil-to-lymphocyte ratio (NLR), monocyte-to-lymphocyte ratio (MLR), and the expression of certain genes (PSMB9, STAT1, and TAP1) [5,12,20]. Moreover, a number of biomarkers, such as IFN-γ, CXCR3, CXCL9, CXCL10, and a combination of miRNAs, have demonstrated notable significance in the diagnosis of subclinical tuberculosis (STB) [14,15]. The findings of this study highlight the potential of utilizing multimodal diagnostic methods that integrate the host immune response and bacterial antigen markers to improve the accuracy of STB diagnosis. Furthermore, the use of biomarkers has yielded valuable insights into the severity of diseases, presenting potential opportunities for customized treatment approaches in the management of spinal TB. Additional mechanistic and clinical investigations are necessary to further enhance the understanding and applicability of these biomarkers in clinical practice.

In a clinical context, spinal tuberculosis symptoms frequently manifest as non-specific symptoms, notably back pain, which may bear resemblance to other musculoskeletal or spinal disorders [1]. Blood tests, such as the measurement of C-reactive protein (CRP) and erythrocyte sedimentation rate (ESR), demonstrate rather limited specificity [33]. The imaging manifestation of spinal tuberculosis has a significant degree of concordance with both other infectious conditions and spinal metastatic cancer [34]. A biopsy is an invasive diagnostic method used to identify spinal tuberculosis and is often performed when the disease is already in the late stage. The complexity associated with diagnosing spinal tuberculosis can result in misdiagnosis or delayed diagnosis, which may contribute to extended patient suffering and an elevated likelihood of consequences, such as spine abnormalities and neurological deficits [2,3].

The identification of potential biomarkers, including NLR, IFN-gamma, and certain combinations of miRNAs, provides clinicians with novel resources to improve the diagnosis of spinal tuberculosis (STB) [5,14]. The utilization of biomarkers has the ability to differentiate spinal tuberculosis (STB) from other spinal diseases, hence mitigating the occurrence of misdiagnosis and expediting early intervention. The incorporation of these markers into clinical practice has the potential to facilitate the evaluation of illness manifestations and customize therapeutic approaches. The integration of numerous biomarkers has the potential to improve the precision of diagnostic assessment. 

Biomarkers such as the monocyte-to-lymphocyte ratio (MLR) and NEAT1 lncRNA (nuclear-enriched abundant transcript 1 long non-coding RNA) have the potential to serve as prognostic indicators, facilitating the implementation of rigorous monitoring and prompt interventions [7,20]. The monitoring of biomarkers during the course of treatment has the potential to yield significant insights into treatment response. The utilization of biomarker-based diagnostics has the potential to enhance the allocation of resources within healthcare settings, thereby potentially leading to a reduction in healthcare expenditures. Subsequent investigations should prioritize external validation of these biomarkers across diverse demographics and healthcare contexts. It is imperative for clinicians to understand the necessity of validation prior to incorporating biomarker-based diagnostics into their clinical practice.

The authors acknowledge the presence of heterogeneity within the studies included in this analysis, which included differences in the populations studied as well as changes in the methodology employed. The presence of heterogeneity emphasizes the necessity of implementing standardized techniques to validate the aforementioned biomarkers. Search keywords are also a limiting factor for search-based studies. Not all researchers in this field use “biomarkers” or “spinal tuberculosis” in their keywords, titles, or conclusions. Furthermore, not all studies provided cutoff, sensitivity, specificity, and AUC values for their biomarkers. This issue obstructed researchers’ efforts in creating a proper meta-analysis. Further studies should report all diagnostic abilities; thus, a proper meta-analysis can be performed. Lastly, using QUADAS-2, we found that most studies were plagued by the lack of an index test, potential bias in patient selection, and a risk of heterogeneity. This might have bias implication, and clinicians should note this issue when considering the use of biomarkers. Further studies with proper design and methodologies may be necessary to create a stronger recommendation.

## 5. Conclusions

Our systematic review revealed a diverse array of biomarkers associated with spinal tuberculosis, showing the complexity of diagnostic possibilities. Notably, the combination of multiple biomarkers, including NLR, PSMB9, STAT1, and specific miRNAs, demonstrated promising diagnostic accuracy. These findings underscore the potential for a multimodal approach utilizing both host immune response markers and bacterial antigens to enhance the precision of spinal tuberculosis diagnosis. Additionally, the identified biomarkers offer insights into disease severity, paving the way for tailored therapeutic interventions for the management of spinal tuberculosis. However, studies in this area are still in the early stages of validation. Further research into the mechanistic roles and clinical utility of these biomarkers is imperative to advance diagnostic and therapeutic strategies for this challenging condition. It may also be necessary for researcher to improve the methodologies and research in this area to increase the applicability of such diagnostic tools.

## Figures and Tables

**Table 1:** Study characteristics and demographics.

Authors	Country	Sample	Reference Standard	Index Test	Population Size	Biomarker Performance
Liu et al., 2022 [5]	China	Blood	Clinical, laboratory, and radiologic evaluations and clinical response to anti-TB drugs or antimicrobial therapy	NLR (neutrophil to lymphocyte ratio)	STB (*n* = 146; male *n* = 78; female *n* = 68; mean age = 55.70 ± 17.16). Control group (PSI) (*n* = 60; male *n* = 34; female *n* = 26; mean age = 63.68 ± 11.52)	NLR cutoff: 6.742 (sensitivity 78.33%, specificity 83.56%)
Qi et al., 2022 [6]	China	Tissue	Culture and histopathology	Mycobacterium tuberculosis-specific antigen/phytohemagglutinin (TBAg/PHA) ratio with AFBS and GeneXpert MTB/RIF	A total of 519 STB (319 subjects at Tongji Hospital (male *n* = 200; female *n* = 119) and 200 subjects at Sino-French New City Hospital (male *n* = 125; female *n* = 75)	TBAg/PHA ratio 0.047 (sensitivity 78.18%, specificity 76.56%) TBAg/PHA ratio combined with AFBS 0.225 (sensitivity 81.82%, specificity 76.56%) TBAg/PHA ratio combined with GeneXpert MTB/RIF 0.171 (sensitivity 83.64%, specificity 76.08%)
Liang et al., 2023 [8]	China	Blood	Clinical manifestations, imaging, laboratory examinations, histopathology	Three-plasma miRNA combination (hsa-miR-506-3p, hsamiR-543, hsa-miR-195-5p)	A total of 423 subjects were recruited with 157 cases of STB, 30 cases of active PTB, 83 cases of SDD, and 153 cases of healthy controls	A three-plasma miRNA combination (hsa-miR-506-3p, hsamiR-543, hsa-miR-195-5p) sensitivity = 80.5%, and specificity = 80.0% Three miRNA signatures could discriminate the STB from other SDD groups with sensitivity = 80%, specificity = 96%, PPV = 84%, NPV = 94%, total accuracy rate = 92%
Zheng et al., 2022 [7]	China	Blood	Imaging, laboratory, histopathology	NEAT1 lncRNA in granulomatous tissue vs peripheral blood	STB (*n* = 120; male *n* = 63; female *n* = 57; age range = 14–91 y; (<60 y *n* = 78; ≥60 y *n* = 42)) Healthy control group (*n* = 60; male *n* = 37; female *n* = 28; age range = 20–80 y)	NEAT1 lncRNA was significantly increased in peripheral blood and granulomatous tissues of STB patients. NEAT1 lncRNA was significantly upregulated in macrophages infected with Mtb, and the difference was statistically significant compared with Mtb-uninfected group. The expression level of NEAT1 lncRNA in granulomatous tissue of STB was significantly higher than that in peripheral blood.
Wu et al., 2023 [12]	China	Tissue	Not specified	Proteasome 20 S subunit beta 9 (PSMB9), signal transducer and activator of transcription 1 (STAT1), and transporter 1 (TAP1)	STB (*n* = 5) Thoracolumbar disk herniation as control group (*n* = 5)	The expression of proteasome 20 S subunit beta 9 (PSMB9), signal transducer and activator of transcription 1 (STAT1), and transporter 1 (TAP1) was found to be particularly high in patients with spinal TB and other extrapulmonary TB as well as in TB and multidrug-resistant TB (*p* < 0.05). They revealed high diagnostic and predictive values and were closely related to the expression of multiple immune cells (*p* < 0.05).
Lou et al., 2022 [13]	China	Blood	Culture and histopathology	LBP	STB (*n* = 100; male *n* = 50; female *n* = 50; mean age = 49.47 ± 16.32 y; age range = 18–77 y). Healthy control group (*n* = 30; male *n* = 13; female *n* = 17, mean age = 53.39 ± 9.67 y; age range = 40–72 y)	LBP protein in peripheral blood is significantly higher in patients with spinal tuberculosis than in the normal population.
Sun et al., 2023 [14]	China	Blood	Not specified	miRNA	STB (*n* = 10; male *n* = 3; female *n* = 7) Control group with disc generation (*n* = 10; male *n* = 3; female *n* = 7)	ROC curve revealed that miR125b-5p is a potential diagnostic biomarker for spinal tuberculosis.
Shang et al., 2021 [15]	China	Tissue	Laboratory, imaging, and histopathology	IFN-gamma, CXCR3, and its ligands (CXCL9 and CXCL10)	STB (*n* = 36; male *n* = 18; female *n* = 18, mean age 43.14 ± 15.36 y; age range 18–77 y). Healthy control group (*n* = 20; male *n* = 10; female *n* = 10; mean age 46.65 ± 11.82 y)	IFN-gamma, CXCR3, CXCL9, and CXCL10 levels in peripheral blood of patients were significantly higher than those in healthy controls.
C. Zhou et al., 2023 [16]	China	Tissue	Laboratory, imaging, and histopathology	MMP9 (matrix metallopeptidase 9) and STAT1 (signal transducer and activator of transcription 1)	STB (*n* = 164; male *n* = 95; female *n* = 69, mean age = 45.16 ± 17.04 y) Control group with lumbar spinal stenosis or disc herniation (*n* = 162; male *n* = 93; female *n* = 69; mean age 47.99 ± 17.21 y)	MMP-9 and STAT1 positivity among patients with STB (*p* < 0.01).
Siregar et al., 2020 [17]	Indonesia	Blood	Not specified	Matrix metalloproteinase-9 (MMP-9)	STB (*n* = 5; male *n* = 1; female *n* = 4; mean age 41.6 ± 18.8 y). Control group with degenerative spinal disease (*n* = 5; male *n* = 3; female *n* = 2; mean age = 44.79 ± 16.98 years)	There were significant differences in serum MMP-9 levels between ST and DSDs with a significance value of 0.002 (*p* < 0.05).
T.N. Mann et al., 2021 [1]	South Africa	Blood	Culture and histopathology	Fibrinogen, CRP, IFN-gamma, NCAM, CRP, ferritin, and CXCL8m GDF-15	STB (*n* = 26; male *n* = 12; female *n* = 14) Control group with mechanical back pain (*n* = 17; male *n* = 7; female *n* = 10)	Five-biomarker signature (sensitivity 100%, with a 95% confidence interval (CI) ranging from 89% to 100%; specificity 100%, with a 95% CI ranging from 84% to 100%).
Lan et al., 2020 [18]	China	Blood	Culture, imaging, and histopathology	ANGPTL-4 (angiopoietin-like protein 4)	STB (*n* = 27; male *n* = 10; female *n* = 17; mean age = 47.33 ± 15.43 y; age range = 18–69 y) Brucellosis spinal (*n* = 17; male *n* = 15; female *n* = 11; mean age was 50.95 ± 13.41 y; age range = 31–72 y)	The positive rate of ANGPTL-4 in TS patients (24/27, 88.89%) was higher than that in BS patients (17/26, 65.83%) (*p* < 0.05).
Daniel, K. and Dunn, R., 2013 [19]	South Africa	Blood	Histopathology, TB culture, and TB PCR	Platelet count	STB (*n* = 160; male *n* = 69; female *n* = 91; mean age = 40.5 y; age range = 13–79 y) Non-STB as control group (*n* = 210; male *n* = 85; female *n* = 125; mean age = 54.5 y; age range = 13–86)	Raised platelet count (sensitivity 52.5%; specificity 86.2%)
Chen et al., 2022 [20]	China	Blood	Histopathology	Monocyte-to-lymphocyte ratio (MLR)	STB (*n* = 247; male *n* = 202; female *n* = 145; mean age = 49.4 ± 17.3 y) Non-STB as a control group (*n* = 353; male *n* = 307; female *n* = 46; mean age = 35.4 ± 10.4 y)	The C-index of the nomogram in the training set and external validation set was 0.801 and 0.861, respectively. Similarly, the AUC was 0.801 in the former and 0.861 in the latter. The net benefit ranged from 0.1 to 0.95 for the former nomogram and 0.02 to 0.99 for the latter nomogram.
Wang et al., 2020 [21]	China	Blood	Histopathology	M1 and M2	Patients with STB (*n* = 36; male *n* = 17; female *n* = 19; mean age = 56.20 ± 5.80 y; age range = 4–77 y) Healthy control group (*n* = 25; male *n* = 12; female *n* = 13; mean age = 44.20 ± 11.50 y)	CD68, iNOS, CD163, IL-10, TNF-α, and IFN-γ were expressed around the tuberculous granuloma. RT-PCR and ELISA results indicated that IL-10, TNF-α, and IFN-γ levels of TB patients were also higher than those of the healthy controls.
